# Dural Tenting in Elective Craniotomies: A Randomized Clinical Trial

**DOI:** 10.1227/neu.0000000000003480

**Published:** 2025-05-01

**Authors:** Lukasz Przepiorka, Katarzyna Wójtowicz, Sławomir Kujawski, Karol Wiśniewski, Ernest Jan Bobeff, Rafał Kruk, Bartłomiej Kulesza, Jan Fortuniak, Adam Mróz, Piotr Dunaj, Maciej Kaspera, Szymon Hoppe, Kamil Krystkiewicz, Katarzyna Kwiecień, Dariusz Szczepanek, Dariusz J. Jaskólski, Piotr Ładziński, Radosław Rola, Jacek Furtak, Tomasz Trojanowski, Andrzej Marchel, Przemysław Kunert

**Affiliations:** * Department of Neurosurgery, Medical University of Warsaw, Warsaw, Poland;; ‡ Department of Exercise Physiology and Functional Anatomy, Ludwik Rydygier Collegium Medicum in Bydgoszcz Nicolaus Copernicus University in Toruń, Bydgoszcz, Poland;; § Department of Neurosurgery and Oncology of the Nervous System, Barlicki University Hospital, Medical University of Lodz, Lodz, Poland;; ‖ Neurosurgery and Pediatric Neurosurgery Department in Lublin, Medical University of Lublin, Lublin, Poland;; ¶ Department of Neurosurgery, Medical University of Silesia, Regional Hospital, Sosnowiec, Poland;; # Faculty of Medicine, Bydgoszcz University of Science and Technology, Bydgoszcz, Poland;; ** Department of Neurosurgery, 10-th Military Research Hospital, Bydgoszcz, Poland;; ‡‡ Current Affiliation: Department of Neurosurgery and Neurooncology, Copernicus Memorial Hospital, Lodz, Poland

**Keywords:** Craniotomy, Dural tenting sutures, Extradural hematoma, Randomized clinical trial

## Abstract

**BACKGROUND AND OBJECTIVES:**

Dural tenting sutures are a well-known neurosurgical technique. However, claims of them preventing extradural hematomas (EDHs) lack evidence-based support. For that reason, we decided to evaluate the noninferiority of routinely not tenting the dura in elective supratentorial craniotomies.

**METHODS:**

A randomized, multicenter, investigator-blinded and participant-blinded, controlled interventional trial with 1:1 allocation. We included adults undergoing elective, supratentorial craniotomies. Not tenting the dura was an intervention, and the control group consisted of patients with at least 3 dural tenting sutures. The primary outcome was the risk of reoperation because of EDH, and secondary outcomes included a selection of clinically relevant outcomes.

**RESULTS:**

We randomized 490 patients into intervention (238, 49%, not tenting the dura) and control (252, 51%, dural tenting) study groups, as per intention-to-treat analysis. Proportions of EDH surgeries in the intervention group were noninferior in comparison with the control group and not significantly different using the intention-to-treat (0.8% and 0.4%, *P* = .98), per-protocol (0.5%, 0.4%, *P* > .99), and as-treated (0.5%, 0.7%, *P* > .99) analyses. There were no significant differences in secondary outcomes: postoperative 30-day mortality (0.8%, 1.2%, *P* > .99), postoperative 30-day readmission (1.7%, 4.4%, *P* = .99), new neurological deficit or deterioration of a previous (19.7%, 15.5%, *P* = .81), cerebrospinal fluid leak (1.3%, 4.4%, *P* > .99), deterioration of postoperative headache over 5 numerical rating scale (4.4%, 2.4%, *P* = .85), epidural collection thickness over 3 mm (90.8%, 87.3%, *P* = .81), and midline shift over 5 mm (7.6%, 4.8%, *P* = .791) in the intervention and control study groups in intention-to-treat analysis. Similarly, secondary outcomes were not different in per-protocol and as-treated analyses. Other than cerebrospinal fluid leaks and EDHs, there were 17 adverse events in the intervention group and 19 in the control group (intention-to-treat analysis, 7.1% and 7.5%, respectively).

**CONCLUSION:**

This trial demonstrates the noninferiority of omitting prophylactic dural tenting for postoperative EDH requiring surgery in elective, supratentorial craniotomies.

**ABBREVIATIONS:**
EDH: extradural hematoma
FDR: false discovery rate
MCAR: Missing Completely At Random
POD: postoperative day
PP: per-protocol
TOST: two one-sided tests.

Introduced by Walter Dandy,^1^ dural tenting sutures are among one of the well-known intraoperative neurosurgical techniques. Yet, the claim of them preventing extradural hematomas (EDHs) lacks evidence-based support. Accordingly, some authors suggested that their routine use may be redundant.^2-5^ Unfortunately, none of the previous studies were properly designed randomized trials.^2^

For that reason, we deemed it necessary to evaluate this surgical technique in an evidence-based manner. Hence, we designed a multicenter, randomized, prospective controlled trial with the aim of determining whether not tenting the dura is not worse than executing the usual method of craniotomy closure, specifically dural tenting. In this article, we report the primary and secondary outcomes of our trial.

## METHODS

This is a report of a randomized, multicenter, investigator-blinded and participant-blinded, controlled interventional trial with 1:1 allocation. We already described its design in detail in the open-access study protocol.^6^ Changes made after commencement include the removal of one of the secondary outcomes: the measurement of the extradural collection volume. This was because of its nonpractical aspect and difficult application in a real-world scenario. In addition, we allowed the third clinical evaluation to be conducted earlier than 5 to 7 days postoperatively if the patient was discharged earlier and also extended the time frame for postoperative computed tomography (CT) scan until the third postoperative day (POD) (instead of the second). Except for the abovementioned, adding participating centers and changing recruitment status, there were no other changes in the study protocol.

This report follows the extension of the CONSORT 2010 statement on reporting of noninferiority and equivalence randomized trials.^7^ This study is registered at http://www.clinicaltrials.gov (NCT03658941), and The Bioethics Committee of the Medical University of Warsaw (the local institutional review board) approved the study protocol (KB/106/2018). The interim monitoring results were presented during the European Association of Neurological Surgeons 2020 conference, and the study was continued.

### Inclusion and Exclusion Criteria

We included adults who had had fulfilled all the following criteria: male or female older than 18 years and younger than 75 years, qualified for an elective supratentorial craniotomy with a diameter of at least 3 cm, Glasgow Coma Scale 15 preoperatively, and modified Rankin scale 0, 1, or 2 preoperatively. We excluded patients in case of coagulation abnormalities before the surgery, revision craniotomy, or skull base surgery. There were no previous trials that had established the efficacy of dural tenting (ie, reference treatment). Written consent was obtained from each participant.

### Intervention

In this trial, not tenting the dura was considered an intervention. This design choice emerged from the fact that dural tenting has been regarded as a neurosurgical standard for decades. The control group consisted of patients with at least 3 dural tenting sutures.

### Clinical and Radiological Evaluation

We performed 3 neurological examinations: the first examination 1 day before surgery, the second 1 day after surgery, and the final between 5 and 7 days after surgery. The final clinical evaluation could have been performed earlier if the subject was discharged before the scheduled evaluation. Each of these visits documented the following neurological examinations: Glasgow Coma Scale, modified Rankin Scale,^8^ headaches according to the numerical rating scale^9^, and the muscle power in each limb according to the Medical Research Council System.^10^

The radiological evaluation included midline shift measurement on the most recent preoperative imaging (either MRI or CT) and the postoperative CT completed according to the trial schedule.

### Primary and Secondary Outcomes

The primary outcome is the risk of reoperation because of EDH (EDH occurrence) evaluated during postoperative hospitalization. Secondary outcomes include:

1. Postoperative 30-day mortality
2. Postoperative 30-day readmission to a neurosurgical or neurological department
3. New neurological deficit or deterioration of a preoperative deficit, as evaluated on POD 5 to 7.
4. Cerebrospinal fluid leak requiring treatment.
5. Increase of intensity of postoperative headaches over 5 on the numerical rating scale from baseline.
6. Extradural collection thickness >3 mm.
7. Midline shift >5 mm.

### Missing Data

Missing data were visualized using the naniar package^11^ and analyzed using the misty package^12^ with Little Missing Completely At Random (MCAR) test. Visual inspection, expert opinion, and result of the MCAR test showed that data were not MCAR. There seemed to be a relationship between the number of missing data in primary and secondary outcomes (eight variables), allocation, and study center (MCAR test result *P* = .01). When the study center was removed from the analysis, MCAR test result was nonsignificant (*P* = .78). The relationship between the study center and missing data is shown in **Supplemental Digital Content 1** (http://links.lww.com/NEU/E772). Nevertheless, missing data in primary and secondary outcomes were less than 0.5% (**Supplemental Digital Content 1**, http://links.lww.com/NEU/E772); therefore, we decided to abstain from applying imputation methods.

### Sample Size and Power Calculation

As noted previously,^6^ an online calculator (Sealed Envelope Ltd. 2012) was used to determine the sample size. Based on the literature review, we assumed that the experimental group would experience 0.7% EDH incidence, whereas the control group was predicted to have 1.4%. A 0.7% noninferiority limit, d, was chosen. Based on primary calculations, it was intended to recruit 1000 patients in each group, for a total of 2000 patients. However, eventually, 490 patients in total were recruited and analyzed. Calculating power for two one-sided tests (TOST) for two proportions using the PowerTOST package for R^13,14^ and assuming alpha = 0.05, the total number of participants in each group = 490, proportions in groups based on the actually gathered data as 0.0045 and 0.0075 for the observed primary outcome, and lower bound for noninferiority of −0.007 indicated power at 100%.

### Statistical Analysis

Continuous data are presented as median (IQR), whereas categorical data are presented as count (%). The Shapiro-Wilk and Levene tests were applied to examine the assumptions of data normality and the equality of variances, respectively. Primary outcome analysis (difference in proportion in reoperation because of EDH in interventional vs control group) was performed using TOST equivalence testing for two proportions applying the TOSTER package for R in jamovi with 0.07 upper equivalence bound.^15,16^ Secondary outcomes were not examined using TOST because there were no prespecified assumptions on prevalence or the noninferiority limit. Associations between qualitative variables were tested using the χ^2^ and Fisher exact tests. Differences between the two groups were assessed using either the Mann-Whitney *U* test or independent t-tests, depending on which assumptions were met. We additionally tested the difference in survival times between independent groups (interventional vs control groups) using Cox regression. Binary value on death (present vs not present) and time until death (expressed in days) were included in the models, without any additional predictors. If the assumption on proportionality of hazards was not met, then coxphw package was used using R, to apply weighted estimation in Cox regression.^17^ All *P* values, except those coming from TOSTs, were corrected by applying the Holm method using the false discovery rate (FDR) estimation package.^18^ *P* values both before and after FDR correction are reported.

### Randomization

According to the study protocol, the allocation sequence with a block size of 100 for each participating center was generated by a statistician using a computer-generated consecutive list for either intervention (no dural tenting) or control (dural tenting). Allocations were concealed in a sealed envelope which was revealed to the surgeon intraoperatively by the anesthesiologist team. This was then kept secret, including surgery and discharge notes. Each participating center had a local investigator who was responsible for all the evaluations and was not aware of the treatment allocation nor whether dural tenting sutures were used . Importantly, it was always at the surgeon's discretion to follow randomization or not, based on the intraoperative conditions. This was performed not to pose additional risks on the patient's health, and, because of the blinding in the study, it remained unknown.

## RESULTS

### Study Description

This article reports patients enrolled in the trial from its commencement in September 2018 until its conclusion in September 2022, which marked the end of the extended time frame approved by the Bioethics Committee of the Medical University (the local Institutional Review Board). Figure presents the CONSORT flow diagram, and Table 1 presents the patients' basic demographic and clinical data. A total of 490 patients were distributed among 5 participating centers (Warsaw, 271, 55%, Lodz, 70, 14%, Lublin, 73, 15%, Sosnowiec, 33, 7%, Bydgoszcz, 43, 9%, **Supplemental Digital Content 2**, http://links.lww.com/NEU/E773). There were no significant differences between the intervention (no dural tenting) and control (dural tenting) groups in intention-to-treat and per-protocol (PP) analyses (Table 2 and **Supplemental Digital Content 3**, http://links.lww.com/NEU/E774, respectively). Similarly, we found no differences in the as-treated analysis, from which one participant is excluded because of unknown status of treatment received (**Supplemental Digital Content 4**, http://links.lww.com/NEU/E775).

**FIGURE.**
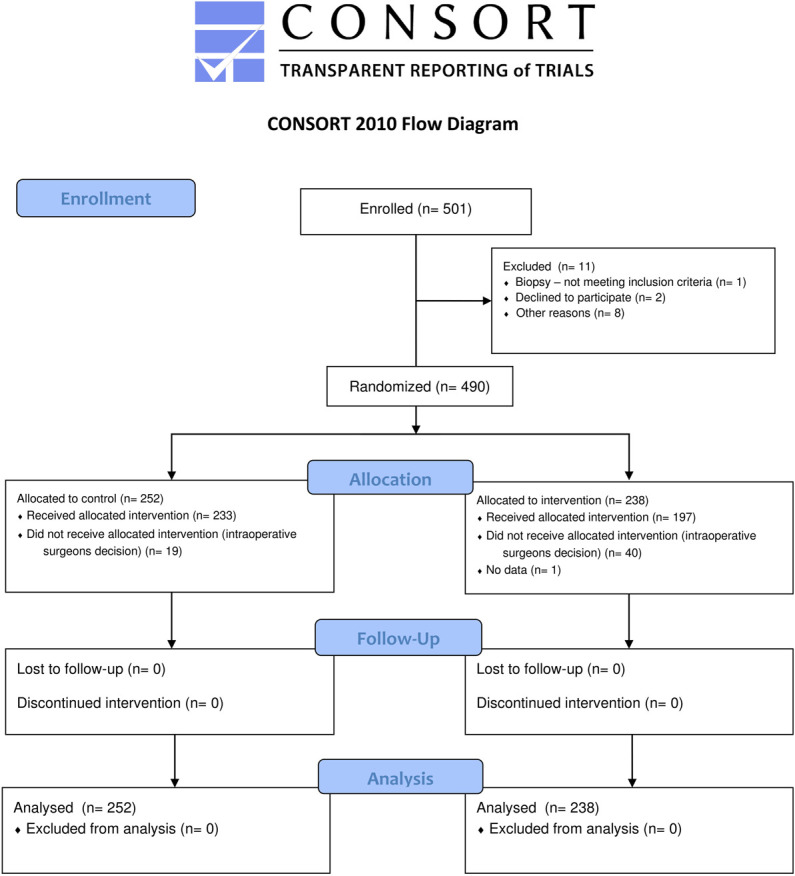
Flow diagram with the intention-to-treat study groups assignment.

**TABLE 1.** Descriptive Characteristics of the Intention-to-Treat Study Group

Characteristic	Intervention^a^	Control^a^	*P* value^b^	*P* value FDR^c^
N	238 (49%)	252 (51%)		
Median age (1st, 3rd quartiles) [y]	55 (41, 65)	57 (41, 65)	.83	>.99
Sex			.52	>.99
Female	114 (48%)	128 (51%)		
Male	124 (52%)	124 (49%)		
Diagnosis			.9	>.99
Arteriovenous malformation	8 (3.4%)	5 (2.0%)		
Aneurysm	11 (4.6%)	13 (5.2%)		
Arachnoid cyst	0	1 (0.4%)		
Brain tumor	203 (84.8%)	217 (85.8%)		
Cavernous malformation	12 (5.0%)	9 (3.6%)		
Colloid cyst	1 (0.4%)	1 (0.4%)		
Dural arteriovenous fistula	2 (0.8%)	4 (1.6%)		
Epilepsy	1 (0.4%)	2 (0.8%)		
Side			>.99	.99
Right	120 (50%)	127 (50%)		
Left	112 (47%)	116 (46%)		
Both/midline	6 (2.5%)	9 (3.6%)		
Comorbidities				
Hypertension				
Yes	78 (33%)	76 (30%)	.53	.96
No	160 (67%)	176 (70%)		
Diabetes				
Yes	21 (8.8%)	14 (5.6%)	.16	>.99
No	217 (91%)	238 (94%)		
Oncological medical history				
Yes	29 (12%)	28 (11%)	.71	>.99
No	209 (88%)	224 (89%)		
Other				
Yes	77 (32%)	75 (30%)	.59	>.99
No	161 (68%)	177 (70%)		

FDR, false discovery rate.

Median (first, third quartiles) or N (%). Percentages are calculated within specific intention-to-treat study group assignment.

^a^Median (IQR); n (%).

^b^Wilcoxon rank sum test; Pearson χ^2^ test; Fisher exact test.

^c^False discovery rate (FDR) correction for multiple testing.

**TABLE 2.** Primary and Secondary Outcomes in Intention-to-Treat Study Group Assignment

Outcome	Intervention (n = 238, 49%)^a^	Control (n = 252, 51%)^a^	*P* value^b^	*P* value FDR^c^
Reoperation because of EDH			.61	.98
Yes	2 (0.8%)	1 (0.4%)		
No	236 (99.2%)	251 (99.6%)		
Postoperative 30-day mortality			>.99	>.99
Yes	2 (0.8%)	3 (1.2%)		
No	236 (99.2%)	248 (98.8%)		
Postoperative 30-day readmission	`		.085	.99
Yes	4 (1.7%)	11 (4.4%)		
No	234 (98.3%)	240 (95.6%)		
New neurological deficit or deterioration			.21	.81
Yes	47 (19.7%)	39 (15.5%)		
No	191 (80.3%)	213 (84.5%)		
Cerebrospinal fluid leak			.039	>.99
Yes	3 (1.3%)	11 (4.4%)		
No	235 (98.7%)	241 (95.6%)		
Deterioration of postoperative headaches over 5 NRS			.19	.85
Yes	10 (4.4%)	6 (2.4%)		
No	216 (95.6%)	240 (97.6%)		
Not testable	2 (0.8%)	0		
No data	10 (4.2%)	6 (2.4%)		
Epidural collection thickness over 3 mm			.25	.81
Yes	216 (90.8%)	219 (87.3%)		
No	22 (9.2%)	32 (12.7%)		
No data	0	1 (0.4%)		
Midline shift over 5 mm			.26	.791
Yes	18 (7.6%)	12 (4.8%)		
No	220 (92.4%)	239 (95.2%)		
No data	0	1 (0.4%)		

EDH, extradural hematoma; FDR, false discovery rate; NRS, numerical rating scale.

^a^n (%).

^b^Fisher exact test; Pearson χ^2^ test.

^c^False discovery rate (FDR) correction for multiple testing.

No significant differences were found in descriptive characteristics and risk factors at the study baseline between intervention (no dural tenting) vs control (dural tenting) study group division (Table 1).

### Primary and Secondary Outcomes—Intention-to-Treat Analysis

Analysis of difference in proportions of primary and secondary outcomes between the groups according to intention-to-treat analysis is presented in Table 2. After correction for FDR, we noticed no significant differences between the study groups.

Z-scores and *P* values for Z-tests, and TOST upper and lower bounds for proportion difference in the presence of EDH reoperation in intention-to-treat study groups are presented in **Supplemental Digital Content 5** (http://links.lww.com/NEU/E776). A nonsignificant result for upper bound (*P* = .054) and significant result for lower bound (*P* < .001) (90% confidence interval for equivalence bounds (lower; higher) (−0.007; 0.016) were noted. Therefore, the proportion of EDH reoperation in intention-to-treat in the intervention study group (no dural tenting) could be considered as noninferior in comparison with the control. The result of the z-test (*P* = .267) suggests that difference in proportion in difference for EDH reoperation rate in the intention-to-treat study group is not significant.

### Primary and Secondary Outcomes—Per-Protocol Analysis

Analysis of difference in proportions of primary and secondary outcomes between the groups according to PP analysis is presented in **Supplemental Digital Content 3** (http://links.lww.com/NEU/E774). After correction for FDR, we noticed no significant differences between the two groups.

Z-scores and *P* values for Z-tests, and TOST upper and lower bounds for the proportion difference for EDH reoperation in PP study groups are presented in **Supplemental Digital Content 6** http://links.lww.com/NEU/E777). A nonsignificant result for upper bound (*P* = .127) and significant result for lower bound (*P* < .001) (90% confidence interval for equivalence bounds (lower; higher) (−0.01; 0.01) were noted. Therefore, the proportion of EDH reoperation in the PP intervention study group (no dural tenting) could be considered as noninferior in comparison with the control. The result of the z-test (*P* = .463) suggests that the difference in proportion of EDH reoperation in the PP study group is not significant. Of note, this analysis includes only two instances of EDH reoperation because the third patient was randomized to the control group; however, the surgeon intraoperatively decided to use dural tenting. Following from this, because of nonadherence to the treatment, this patient was removed from PP analysis.

### Primary and Secondary Outcomes—Summary

**Supplemental Digital Content 7** (http://links.lww.com/NEU/E778) shows the results of TOST for two proportions in EDH reoperation according to intention-to-treat (panel A) and PP (panel B) analyses. One-sided tests for lower bounds provided *P* values <.05 in intention-to-treat and PP analyses. Similarly, the Fisher exact test resulted in *P* values >.05. Based on these two findings and relative and absolute occurrence of the primary outcome in both groups, we conclude that proportions of EDH surgeries in the intervention (no dural tenting) and control (dural tenting) study groups are noninferior and not practically or statistically significantly different.

**Supplemental Digital Content 8** (http://links.lww.com/NEU/E779) shows the number of patients with primary and secondary outcomes in intention-to-treat (panel A) and PP (panel B) analyses. Epidural thickness <3 mm instead of >3 mm was presented on this figure to increase its visibility.

### Neurological and Radiological Outcomes

We compared basic neurological data evaluated at different points of trial participants as indicated in the trial protocol, according to the intention-to-treat analysis. There were no significant differences in comparable parameters between the two groups (Table 3, *P*- and q values are not shown in the table for the purpose of clarity).

**TABLE 3.** Clinical Characteristics of the Intention-to-Treat Study Groups

Characteristic	Intervention group, n = 238 (49%)			Control group, n = 252 (51%)		
	Before surgery	POD 1	POD 5-7	Before surgery	POD 1	POD 5-7
Headache, NRS						
Median (Q1, Q3)	0 (0, 2)	2 (0, 4)	1 (0, 3)	0 (0, 3)	2 (0, 4)	1 (0, 3)
No data (n)	2 (0.8%)	4 (1.7%)	10 (4.2%)	0	1 (0.4%)	8 (3.2%)
Not testable (n)	0	6 (2.5%)	5 (2.1%)	0	3 (1.2%)	1 (0.4%)
GCS						
15	237 (99.6%)	218 (92%)	224 (94%)	252 (100%)	244 (97%)	249 (99%)
14	0	13 (5.5%)	8 (3.4%)	0	7 (2.8%)	3 (1.2%)
13	0	2 (0.8%)	3 (1.3%)	0	1 (0.4%)	0
12	0	2 (0.8%)	0	0	0	0
7	0	1 (0.4%)	1 (0.4%)	0	0	0
3	0	1 (0.4%)	1 (0.4%)	0	0	0
No data	1 (0.4%)	1 (0.4%)	1 (0.4%)	0	0	0
mRS						
0	175 (74%)	120 (50%)	141 (59%)	174 (69%)	129 (51%)	151 (60%)
1	43 (18%)	62 (26%)	54 (23%)	59 (23%)	74 (29%)	70 (28%)
2	19 (8.0%)	21 (8.8%)	19 (8.0%)	18 (7.1%)	27 (11%)	19 (7.5%)
3	0	19 (8.0%)	14 (5.9%)	1 (0.4%)	10 (4.0%)	5 (2.0%)
4	0	12 (5.0%)	7 (2.9%)	0	12 (4.8%)	7 (2.8%)
5	0	3 (1.3%)	2 (0.8%)	0	0	0
No data	1 (0.4%)	1 (0.4%)	1 (0.4%)	0	0	0
Motor deficit						
Present	24 (10%)	39 (16%)	34 (14%)	35 (14%)	48 (19%)	38 (15%)
Absent	213 (89%)	197 (83%)	202 (85%)	217 (86%)	204 (81%)	214 (85%)
Not testable	0	1 (0.4%)	1 (0.4%)	0	0 (0%)	0 (0%)
No data	1 (0.4%)	1 (0.4%)	1 (0.4%)	0	0 (0%)	0 (0%)
Speech deficit						
Present	17 (7.1%)	34 (14%)	38 (16%)	21 (8.3%)	30 (12%)	32 (13%)
Absent	220 (92%)	201 (84%)	197 (83%)	231 (92%)	222 (88%)	220 (87%)
No data	1 (0.4%)	1 (0.4%)	1 (0.4%)	0	0	0
Not testable	0	2 (0.8%)	2 (0.8%)	0	0	0
Other deficits						
Present	24 (10%)	42 (17.5%)	37 (15.4%)	26 (10%)	42 (16.7%)	37 (14.7%)
Absent	213 (89%)	195 (82%)	201 (84%)	226 (90%)	210 (83%)	215 (85%)
No data	1 (0.4%)	1 (0.4%)	1 (0.4%)	0	0	0

GCS, Glasgow Coma Scale; mRS, modified Rankin scale; NRS, numerical rating scale; POD, postoperative day.

There were no significant differences in comparable parameters between the two groups (*P* and q values not shown in the table).

Similarly, there were no differences between the basic radiological preoperative and postoperative parameters (Table 4). Table 4 presents analysis of difference in radiological characteristics of the intervention (no dural tenting) vs control (dural tenting) groups according to intention-to-treat protocol. Before and after correction for FDR, we noticed no significant differences between the two study groups.

**TABLE 4.** Radiological Characteristics of the Intention-to-Treat Study Group

Characteristic	Intervention group, n = 238 (49%)^a^	Control group, n = 252 (51%)^a^	*P* value^b^	*P* value FDR^c^
Preoperative midline shift (mm)			.44	>.99
Median (Q1, Q3)	1.6 (0.0, 4.2)	1.6 (0.0, 5.4)		
No data	11	9		
Postoperative midline shift (mm)			.91	>.99
Median (Q1, Q3)	2.95 (1.35, 4.86)	2.90 (1.40, 4.68)		
No data	0	1		
Extradural collection thickness (mm)			.093	.9
Median (Q1, Q3)	5.65 (4.25, 7.25)	5.20 (4.07, 7.03)		
No data	0	1		
Craniotomy area (cm^2^)			.69	>.99
Median (Q1, Q3)	28 (19, 39)	27 (18, 41)		
No data	0	3		

FDR, false discovery rate.

^a^Median (IQR).

^b^Wilcoxon rank sum test.

^c^False discovery rate correction for multiple testing.

### Kaplan-Meier Analysis of Study Participant Outcomes

**Supplemental Digital Content 9** (http://links.lww.com/NEU/E780) shows a Kaplan-Meier curve displaying the time until death for all patients in whom it was recorded (n = 419 as of February 27, 2024, panel A) by the intervention (no dural tenting) vs control (dural tenting) group according to the intention-to-treat (panel B) and PP (panel C) analyses. No statistically significant differences between the control vs intervention study groups were noted in the time until death according to intention-to-treat (coefficient = 0.19, *P* = .33, panel A) and PP (coefficient = 0.28, *P* = .19, panel B) analyses. In the intention-to-treat analysis, there were 79 deaths in the intervention group (no dural tenting) of 206 patients and 90 deaths in the control group (dural tenting) of 216 patients. In the PP analysis, there were 67 deaths in the intervention group (no dural tenting) of 178 patients and 85 deaths in the control group (dural tenting) of 196 patients.

### Harms

Other than cerebrospinal fluid leaks and EDHs, there were 17 adverse events in the intervention group (no dural tenting) and 19 in the control group (dural tenting), as per intention-to-treat analysis (7.1% and 7.5%, respectively, **Supplemental Digital Content 10**, http://links.lww.com/NEU/E781). There was additionally one case of a traumatic EDH that occurred after discharge of the patient, on the 11th POD. Notably, before that, the patient was diagnosed with epilepsy and antiepileptic drugs were initiated. Despite that, he had an epileptic seizure and trauma, which caused EDH and required surgical evacuation. However, this was deemed as not meeting the primary outcome of reoperation of EDH because of its traumatic nature.

## DISCUSSION

Postoperative EDH is a potentially catastrophic complication after an elective craniotomy: it often requires reoperation, is associated with additional morbidity and mortality, and prolongs hospitalization. The heavy burden of these complications increases even further when it postpones subsequent oncological treatment, for example, in patients with glioma or brain metastases. A common neurosurgical belief states that dural tenting sutures prevent postoperative EDH. In this trial, we found that the proportion of EDH surgeries in the intervention group (no dural tenting) was noninferior in comparison with the control group and not significantly different when using intention-to-treat, PP, or as-treated analyses. In other words, this study provides high-quality evidence that not placing prophylactic dural tenting sutures is not inferior to the current standard of care, which traditionally involved dural tenting.

Our study was designed to evaluate the most important clinical aspect of postoperative EDH, which is articulated with a primary outcome that focuses on reoperation to evacuate EDH. We deemed this as the most clinically significant question to be answered with this trial. Secondary outcomes were designed to evaluate other aspects of clinical importance. The 30-day mortality and 30-day readmission rates provide insight into immediate and short-term outcomes after elective supratentorial craniotomies. Similarly, evaluation of neurological deficits was used to measure short-term functional outcomes. None of these secondary outcomes was significantly different between the two study groups.

We also hypothesized that dural suturing may increase postoperative headaches, as the dura is a pain-sensitive structure. On the other hand, extradural collection thickness may also stretch the dura and contribute to postoperative headaches. What we found was that, yet again, postoperative headaches were not different between the two groups. Interestingly, dural tenting sutures were associated with higher cerebrospinal fluid leak ratios. Although there is a theoretical mechanism in which dural sutures cause holes in the dura, this difference was not statistically significant.

Finally, our secondary radiological outcomes, namely extradural collection thickness and midline shift measurements are easily reproducible and are fundamental radiological parameters. These may influence the decision-making process when evaluating patients and their imaging after surgery. In our trial, however, both extradural collection thickness and midline shift were not different.

The clinical significance of our novel results lies in the potential shortening of surgeries and reducing unnecessary, risky maneuvers that are involved in dural tenting. Multicenter design of our trial allows for generalizability of these results.

Neurosurgery suffers from insufficient evidence-based guidelines in comparison with other specialties. Randomized trials constitute a crucial and irreplaceable aspect of clinical research. Despite their complexity, they are able to prove the redundancy of even the most basic neurosurgical techniques. Our findings challenge a common neurosurgical practice. This opens discussion into the scientific evaluation of other neurosurgical techniques.

### Limitations

Our a priori calculation of sample size indicated that 1816 patients in total required to be analyzed to obtain 90% power. By contrast, the above results are presented based on 490 patients in total. That said, the above study could not be regarded as underpowered because post hoc power calculations indicated 100% power.

## CONCLUSION

This trial demonstrates the noninferiority of omitting prophylactic dural tenting for EDH requiring surgery in adults undergoing elective, supratentorial craniotomies. In addition, proportions of postoperative 30-day mortality and readmission, neurological deficits, increase of intensity of postoperative headaches, extradural collection thickness over 3 mm, and midline shift over 5 mm were not significantly different between the two study groups.

## Supplementary Material

SUPPLEMENTARY MATERIAL

## SUPPLEMENTAL DIGITAL CONTENT

**Supplemental Digital Content 1. Figure 1.** Illustration of missing data in allocation and primary and secondary outcomes. Panel A shows presence of missing data in primary and secondary outcomes in relation with the study site. Missing data are shown in dark gray in both panels. Panel B shows count of missing data in primary and secondary outcomes in respect to the study site. **Supplemental Digital Content 2. Figure 2.** A figure illustrating participating centers along with the number and percentage of trial participants. **Supplemental Digital Content 3. Table 1.** Primary and secondary outcomes in PP study groups. ^1^n (%); ^2^Fisher exact test; Pearson χ^2^ test; ^3^FDR correction for multiple testing. **Supplemental Digital Content 4. Table 2.** Primary and secondary outcomes in as-treated study groups. ^1^n (%); ^2^Fisher exact test; Pearson χ^2^ test; ^3^FDR correction for multiple testing. **Supplemental Digital Content 5. Table 3.** TOST results for proportion difference for EDH operation in intention-to-treat study group assignment. **Supplemental Digital Content 6. Table 4.** TOST results for proportion difference for EDH reoperation in PP study group assignment. **Supplemental Digital Content 7. Figure 3.** Results of TOST for two proportions in EDH surgeries according to intention-to-treat (panel A) and PP (panel B) assignments. **Supplemental Digital Content 8. Figure 4.** Count of occurrence of primary and secondary outcomes in the intervention group (orange) and control (blue). Panel A shows grouping according to intention-to-treat (ITT) analysis, whereas panel B represents grouping according to PP analysis. The panel on the right-hand side shows the difference in occurrence between intervention and control study groups. **Supplemental Digital Content 9. Figure 5.** Kaplan-Meier curve showing time until death for all patients (panel A) in intervention vs control groups according to intention-to-treat (ITT, panel B) and PP (panel C) analyses. **Supplemental Digital Content 10. Table 5.** Adverse events excluding postoperative EDH and cerebrospinal fluid leak in the intention-to-treat study groups. Percentages are calculated within the intention-to-treat study groups. False discovery rate (FDR) correction for multiple testing.
