# Supplementary figures and images for: Dural Tenting in Elective Craniotomies: A Randomized Clinical Trial
Source: Neurosurgery. 2025 May 1;97(5):1108–17. doi: 10.1227/neu.0000000000003480 (PMC12507126; doi:10.1227/neu.0000000000003480)

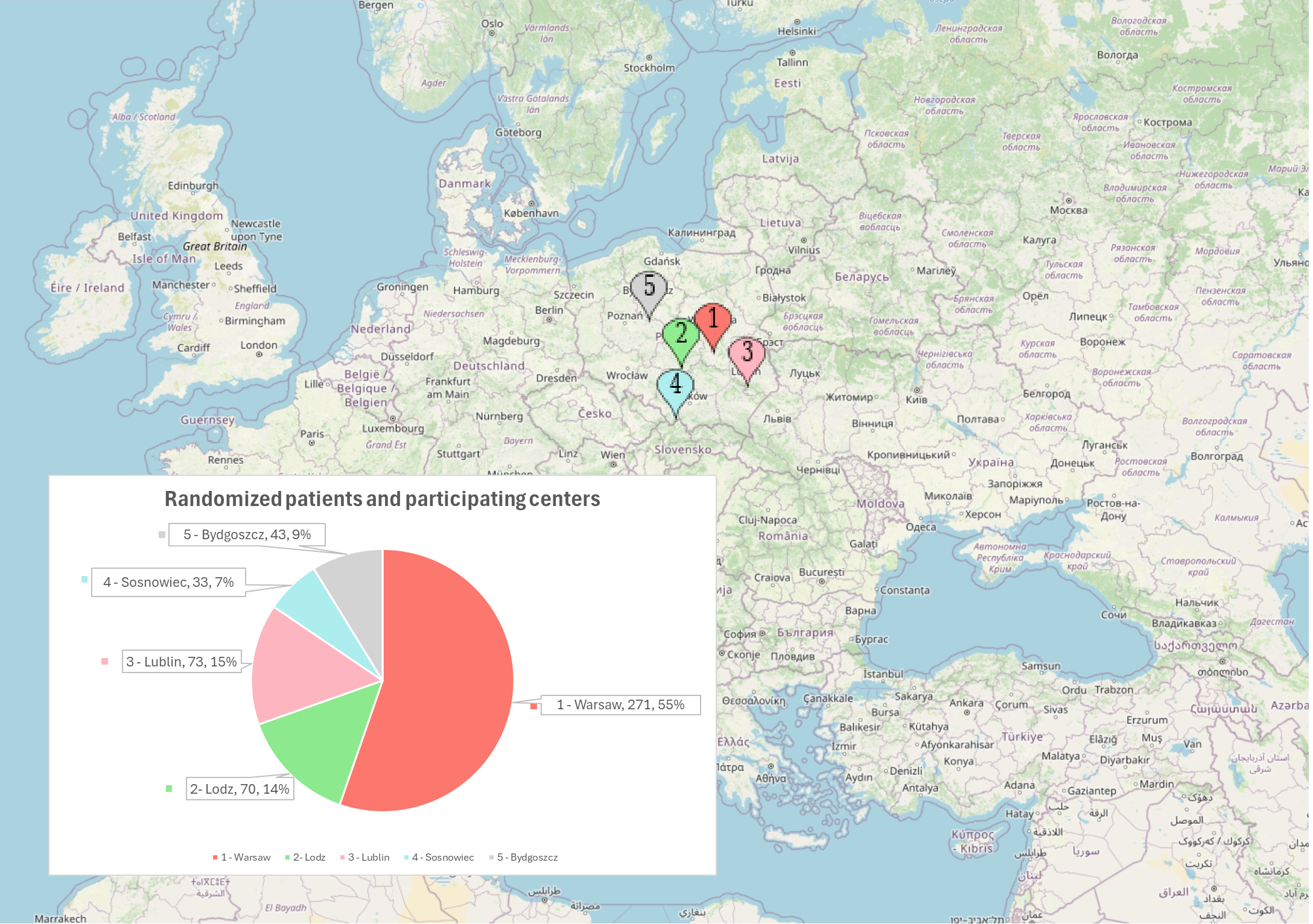

Supplement: SUPPLEMENTARY MATERIAL [file neu-97-1108-s003.tiff]

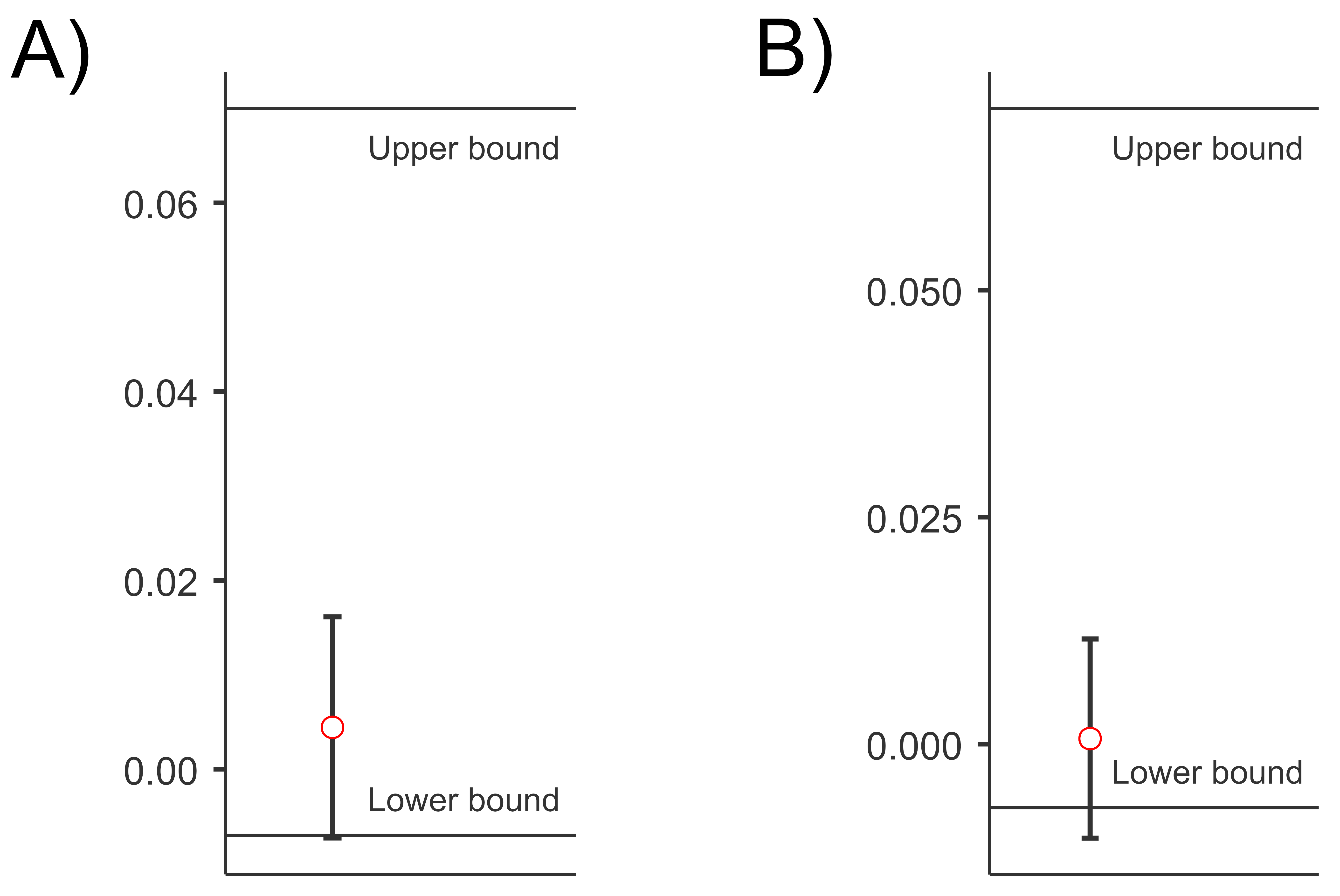

Supplement: SUPPLEMENTARY MATERIAL [file neu-97-1108-s004.tif]

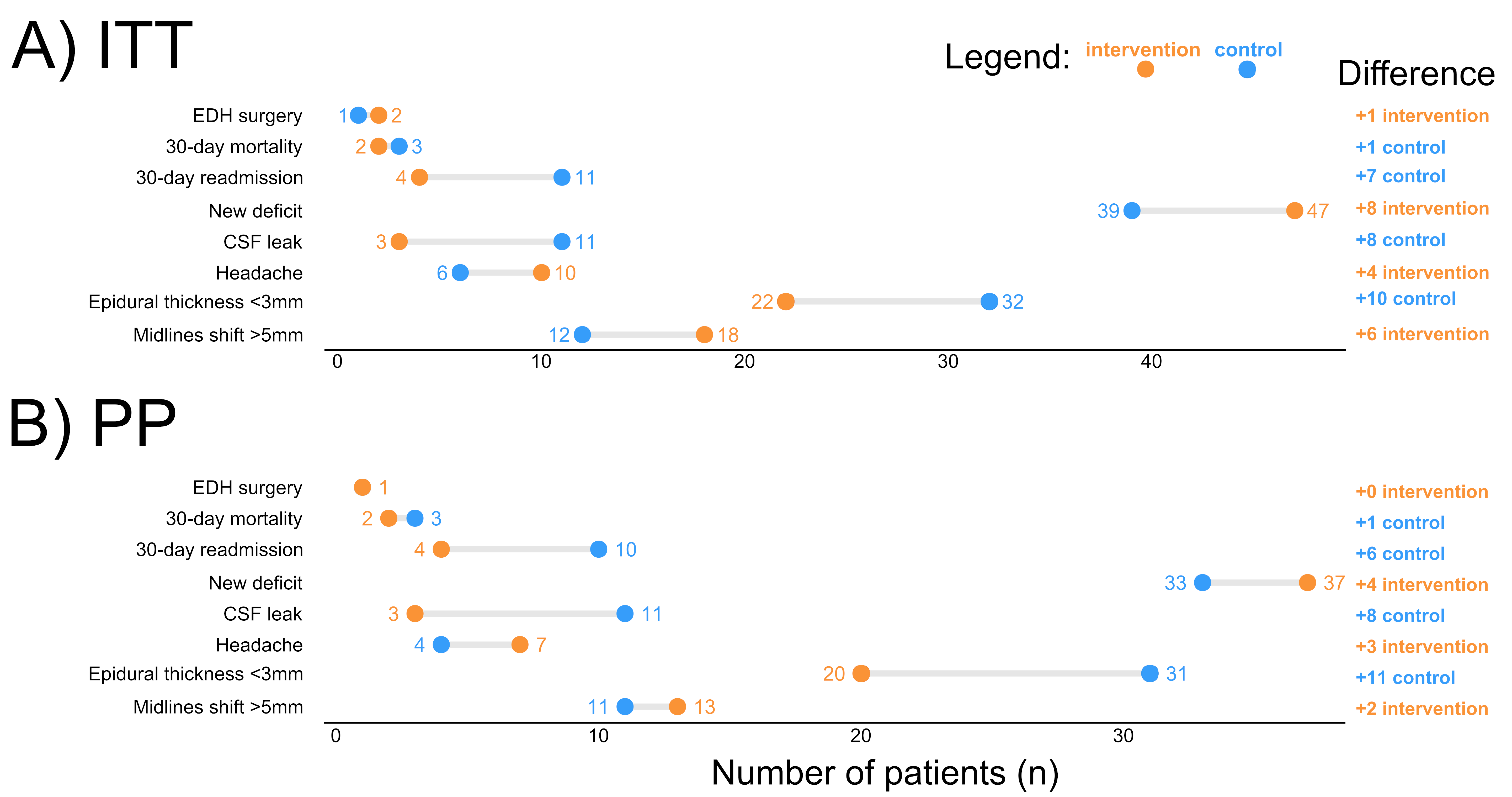

Supplement: SUPPLEMENTARY MATERIAL [file neu-97-1108-s005.tif]
