# Supplementary material for: Dural Tenting in Elective Craniotomies: A Randomized Clinical Trial
Source: Neurosurgery. 2025 May 1;97(5):1108–17. doi: 10.1227/neu.0000000000003480 (PMC12507126; doi:10.1227/neu.0000000000003480)
Supplement: SUPPLEMENTARY MATERIAL [file neu-97-1108-s007.docx]

**Supplementary Table 1. Primary and secondary outcomes in per-protocol study groups.**

| **Outcome** | **Intervention  (n = 199, 47%)** | **Control**  **(n = 227, 53%)** | **p–value** | **p–value FDR** |
| --- | --- | --- | --- | --- |
| **Reoperation due to EDH** |  |  | >0.99 | >0.99 |
| yes | 1 (0.5%) | 1 (0.4%) |  |  |
| no | 198 (99%) | 226 (100%) |  |  |
| **Postoperative 30-day mortality** |  |  | >0.99 | >0.99 |
| yes | 2 (1.0%) | 3 (1.3%) |  |  |
| no | 197 (99%) | 224 (99%) |  |  |
| **Postoperative 30-day readmission** |  |  | 0.17 | 0.9 |
| yes | 4 (2.0%) | 10 (4.4%) |  |  |
| no | 195 (98%) | 217 (96%) |  |  |
| **New neurologic deficit or deterioration** |  |  | 0.26 | 0.75 |
| yes | 37 (19%) | 33 (15%) |  |  |
| no | 162 (81%) | 194 (85%) |  |  |
| **Cerebrospinal fluid leak** |  |  | 0.054 | 0.78 |
| yes | 3 (1.5%) | 11 (4.8%) |  |  |
| no | 196 (98%) | 216 (95%) |  |  |
| **Deterioration of postoperative headaches over 5 NRS** |  |  | 0.04 | 0.77 |
| yes | 7 (3.5%) | 4 (1.8%) |  |  |
| no | 181 (91%) | 220 (97%) |  |  |
| not testable | 2 (1.0%) | 0 (0%) |  |  |
| no data | 9 (4.5%) | 3 (1.3%) |  |  |
| **Epidural collection thickness over 3 mm** |  |  | 0.30 | 0. 83 |
| yes | 179 (90%) | 196 (86%) |  |  |
| no | 20 (10%) | 31 (14%) |  |  |
| **Midline shift over 5 mm** |  |  | 0.53 | 0.93 |
| yes | 13 (6.5%) | 11 (4.8%) |  |  |
| no | 186 (93%) | 216 (95%) |  |  |

^1^ n (%); ^2^ Fisher's exact test; Pearson's Chi-squared test; ^3^ False discovery rate (FDR) correction for multiple testing.
