# Supplementary material for: Dural Tenting in Elective Craniotomies: A Randomized Clinical Trial
Source: Neurosurgery. 2025 May 1;97(5):1108–17. doi: 10.1227/neu.0000000000003480 (PMC12507126; doi:10.1227/neu.0000000000003480)
Supplement: SUPPLEMENTARY MATERIAL [file neu-97-1108-s008.docx]

**Supplementary table 2. Primary and secondary outcomes in as-treated study groups.**

| **Outcome** | **Intervention  (n = 222, 45%)** | **Control**  **(n = 267, 55%)** | **p–value** | **q–value** |
| --- | --- | --- | --- | --- |
| **Reoperation due to EDH** |  |  | >0.99 | >0.99 |
| yes | 1 (0.5%) | 2 (0.7%) |  |  |
| no | 221 (100%) | 265 (99%) |  |  |
| **Postoperative 30-day mortality** |  |  | >0.99 | >0.99 |
| yes | 2 (0.9%) | 3 (1.1%) |  |  |
| no | 220 (99%) | 264 (99%) |  |  |
| **Postoperative 30-day readmission** |  |  | 0.34 | 0.55 |
| yes | 5 (2.3%) | 10 (3.7%) |  |  |
| no | 217 (98%) | 257 (96%) |  |  |
| **New neurologic deficit or deterioration** |  |  | 0.35 | 0.55 |
| yes | 43 (19%) | 43 (16%) |  |  |
| no | 179 (81%) | 224 (84%) |  |  |
| **Cerebrospinal fluid leak** |  |  | 0.068 | 0.27 |
| yes | 3 (1.4%) | 11 (4.1%) |  |  |
| no | 219 (99%) | 256 (96%) |  |  |
| **Deterioration of postoperative headaches over 5 NRS** |  |  | 0.014 | 0.11 |
| yes | 9 (4.1%) | 6 (2.2%) |  |  |
| no | 199 (90%) | 257 (96%) |  |  |
| not testable | 2 (0.9%) | 0 |  |  |
| no data | 12 (5.4%) | 4 (1.5%) |  |  |
| **Epidural collection thickness over 3 mm** |  |  | 0.31 | 0.55 |
| yes | 200 (90%) | 234 (88%) |  |  |
| no | 21 (9.5%) | 33 (11%) |  |  |
| no data | 1 (0.5%) | 0 |  |  |
| **Midline shift over 5 mm** |  |  | 0.70 | 0.93 |
| yes | 14 (6.3%) | 16 (6.0%) |  |  |
| no | 207 (93%) | 251 (94%) |  |  |
| no data | 1 (0.5%) | 0 |  |  |

^1^ n (%); ^2^ Fisher's exact test; Pearson's Chi-squared test; ^3^ False discovery rate correction for multiple testing
