# Supplementary material for: Dural Tenting in Elective Craniotomies: A Randomized Clinical Trial
Source: Neurosurgery. 2025 May 1;97(5):1108–17. doi: 10.1227/neu.0000000000003480 (PMC12507126; doi:10.1227/neu.0000000000003480)
Supplement: SUPPLEMENTARY MATERIAL [file neu-97-1108-s010.docx]

**Supplementary Table 4.** **Two One-Sided Tests (TOST) results for proportion difference for extradural hematoma reoperation in per-protocol study groups assignment.**

|  | **Z** | **p** |
| --- | --- | --- |
| **Z-test** | 0.09 | 0.463 |
| **TOST Upper bound** | 1.14 | 0.127 |
| **TOST Lower bound** | -10.41 | <0.001 |
