# Supplementary material for: Dural Tenting in Elective Craniotomies: A Randomized Clinical Trial
Source: Neurosurgery. 2025 May 1;97(5):1108–17. doi: 10.1227/neu.0000000000003480 (PMC12507126; doi:10.1227/neu.0000000000003480)
Supplement: SUPPLEMENTARY MATERIAL [file neu-97-1108-s011.docx]

**Supplementary Table 5. Adverse events excluding postoperative extradural hematoma and cerebrospinal fluid leak in the intention-to-treat study groups.**

| **Intervention group, n = 238 (49%)** | | **Control group, n = 252 (51%)** | | **p-value** | **p-value FDR** |
| --- | --- | --- | --- | --- | --- |
| **Event** | **Count, n (%)** | **Event** | **Count, n (%)** |  |  |
| intracerebral hematoma in the cavity | 7 (2.9%) | intracerebral hematoma in the cavity | 10 (4%) | 0.53 | 0.99 |
| intraparenchymal hematoma | 2 (0.8%) | intraparenchymal hematoma | 1 (0.4%) | 0.61 | >0.99 |
| subdural hematoma | 1 (0.4%) | subdural hematoma | 2 (0.8%) | >0.99 | >0.99 |
| wound abscess | 2 (0.8%) | wound abscess | 0 | 0.24 | 0.87 |
| intraoperative brain swelling | 1 (0.4%) | intraoperative brain swelling | 0 | 0.49 | >0.99 |
| intraoperative bleeding and cardiopulmonary arrest | 1 (0.4%) | intraoperative bleeding and cardiopulmonary arrest | 0 | 0.49 | >0.99 |
| ischemic stroke | 1 (0.4%) | ischemic stroke | 0 | 0.49 | 0.98 |
| partial tumor removal | 1 (0.4%) | partial tumor removal | 0 | 0.49 | >0.99 |
| venous stroke | 1 (0.4%) | venous stroke | 0 | 0.49 | >0.99 |
| traumatic extradural hematoma | 0 | traumatic extradural hematoma | 1 (0.4%) | >0.99 | >0.99 |
| hydrocephalus | 0 | hydrocephalus | 1 (0.4%) | >0.99 | >0.99 |
| septic shock, wound dehiscence | 0 | septic shock, wound dehiscence | 1 (0.4%) | >0.99 | >0.99 |
| steroid psychosis | 0 | steroid psychosis | 1 (0.4%) | >0.99 | >0.99 |
| venous thrombosis | 0 | venous thrombosis | 1 (0.4%) | >0.99 | >0.99 |
| wound dehiscence | 0 | wound dehiscence | 1 (0.4%) | >0.99 | >0.99 |

Percentages are calculated within the intention-to-treat study groups. False discovery rate (FDR) correction for multiple testing
